# US Public Health and Biomedical Research Institutions Help Keep the World Safe and Deserve Continued Strong Support

**DOI:** 10.4269/ajtmh.25-0156

**Published:** 2025-03-13

**Authors:** Philip J. Rosenthal, Davidson H. Hamer, Daniel G. Bausch, Jamie Bay Nishi, David A. Fidock

**Affiliations:** ^1^ Department of Medicine, University of California, San Francisco, California;; ^2^ Editor-in-Chief, American Journal of Tropical Medicine and Hygiene, Arlington, Virginia;; ^3^ Department of Global Health, Boston University School of Public Health and Department of Medicine, Boston University Chobanian and Avedisian School of Medicine, Boston, Massachusetts;; ^4^ Scientific Program Chair, American Society of Tropical Medicine and Hygiene (ASTMH), Arlington, Virginia;; ^5^ Center for Infectious Disease Emergency Response, National University of Singapore;; ^6^ London School of Hygiene and Tropical Medicine, London, United Kingdom;; ^7^ Past President, ASTMH, Arlington, Virginia;; ^8^ Chief Executive Officer, ASTMH, Arlington, Virginia;; ^9^ Departments of Microbiology and Immunology and of Medicine, Columbia University Irving Medical Center, New York, NY; President, ASTMH, Arlington, Virginia

The American Society of Tropical Medicine and Hygiene (ASTMH) is the largest international scientific organization of experts dedicated to reducing the worldwide burden of tropical infectious diseases and improving global health. In 2020, our leadership expressed serious concerns regarding political interference with essential American programs whose mission is to improve global public health and advance research on our most important international health problems.^1^ Now, five years later, we are again compelled to speak out in support of vital public health programs and research that enhances our ability to counter global health threats.

On its first day in office, January 20, 2025, the new US Administration announced withdrawal of the United States from the World Health Organization (WHO). This was the second such announcement, following an initial attempt, in May 2020, during the Administration’s first term, a decision that was later rescinded by the following Administration. The WHO, supported by its 194 member states and operating with a budget smaller than those of some US hospitals, coordinates international health policy, performs global disease surveillance, develops public health guidelines, and leads disease control and eradication programs. Although burdened by its limited control of national health programs and bureaucratic constraints, the WHO has been remarkably successful. It played a pivotal role in the eradication of smallpox, led successful efforts to control many other infectious diseases, and is the key international agency to address recent international disease outbreaks. The decision to leave the WHO is not immediate. Under US law, a one-year notice is required, and so without a change in policy, the United States will leave the WHO in January 2026. We strongly recommend reconsideration of this decision.

The WHO has been a key partner in combatting multidrug-resistant tuberculosis, HIV infection, and malaria; limiting cervical cancer by preventing human papillomavirus infection; and controlling polio and other viral diseases – each of these remains a major risk worldwide, including within the borders of the United States. During the COVID-19 pandemic, the WHO played a major role in early dissemination of key research findings and surveillance data; oversaw the rapid development of reliable diagnostic tests, vaccines, and therapeutics with international partners; and regularly updated evidence-based guidance for control of the pandemic. WHO coordination, combined with strong national programs, helped to curb spread of this highly transmissible viral pathogen in many countries. Although the international impact of COVID-19 was profound, with millions of deaths and massive societal, educational, and economic disruptions, the impacts would undoubtedly have been even greater without the concerted efforts of the WHO. More recent outbreaks have arguably been noteworthy in that their potential magnitude was contained; WHO has led actions to control repeated outbreaks of such dangerous diseases as mpox, Ebola, and Marburg.

Recent US Administration executive orders and policy actions have also had profound impacts on American agencies with critical roles in international public health and research. Most striking for public health is the abrupt dismantling of the United States Agency for International Development (USAID), which makes up less than 1% of the federal budget, and its associated programs–notably the President’s Emergency Program for AIDS Relief (PEPFAR) and the President’s Malaria Initiative (PMI). These are some of the most impactful programs supporting public health worldwide. Programs managed by USAID have been almost entirely shuttered, including many that initially received waivers for lifesaving and humanitarian care. Nearly all USAID employees received termination notices on February 23. International programs have been paralyzed by stop work orders pending a 90-day review for effectiveness, yet the criteria for program evaluation have not been disclosed. Over 5,000 USAID grants and contracts have been cancelled. These decisions directly affect activities of PEPFAR and PMI, which have all but collapsed, with uncertain futures. The impacts of these changes have been profound. Among many examples of immediate effects, antiretroviral drugs will be increasingly unavailable, leading to illness and deaths in those infected with HIV, and increased transmission of infection to infants and adults; the incidence of preventable and potentially lethal childhood diseases will increase with disruptions in immunizations for infections including measles and polio; effective malaria control measures, including insecticide-treated bednets, spraying of houses with insecticides, and utilization of the best drugs for treatment and prevention will be halted, leading to many preventable malaria deaths, mostly in young children; studies to improve treatments for tuberculosis, one of the greatest killers worldwide, will be halted; drugs and diagnostics for treatment of neglected tropical diseases will expire in US warehouses; and access to food for millions of at-risk individuals in the least advantaged parts of the world will be cut-off, leading to increased rates of acute malnutrition and its consequences, including situations where needed food is locked in nearby warehouses with no one to distribute it.

The immediate impacts of the halt in USAID activities are heartbreaking, with limited savings to the US taxpayer, but it is important to also consider how these will affect the safety of Americans. Outbreaks of dangerous infectious diseases, including Ebola, measles, mpox, and avian influenza are occurring now. Numerous arboviruses, including dengue, chikungunya and Oropouche viruses, threaten Americans within the United States and when they travel, exacerbated by the recent slashing of national and international surveillance and crisis response activities. Other outbreaks, including potentially massive pandemics as seen with HIV infection and COVID-19 in recent memory, can be expected. Recent government decisions mean that established routine surveillance, led by CDC, whose activities have been severely disrupted, and WHO, which is slated to lose US support, has been interrupted. Rising egg prices in the United States, due primarily to culling of domestic chickens for avian influenza, are now causing financial distress, but this concern will pale in comparison to potentially huge impacts on human health if the virus evolves to become capable of human-to-human transmission.

The CDC, the primary US public health agency, which is respected and emulated around the world, has also been greatly affected by recent government decisions, with many positions eliminated and key programs on hold. In the United States, the CDC plays an essential role in disease surveillance, including identifying food-borne and other illnesses, offering consultation for US physicians and patients on unusual infections, and providing reference diagnostic capacities for a large range of infectious diseases. The disease outbreaks that the CDC is ideally equipped to assess and act upon continue, from international outbreaks of Ebola and mpox to domestic outbreaks of measles and avian influenza. It is deeply troubling that these changes are occurring on the heels of the greatest pandemic of respiratory viruses in the United States in a century, with over a million deaths from COVID-19. Strengthening trust in public health as we continue to tackle emerging and enduring health challenges is essential. A vibrant CDC is needed now more than ever.

Another major concern is sudden cuts in congressionally authorized and approved funding for the National Institutes of Health (NIH). The recent decision to cap indirect cost compensation to research institutions at much lower levels than has been standard, which is currently stayed pending court consideration, has the potential to tremendously impact the productivity of American medical research. The firing of NIH scientists, whose laboratories include our next generation of innovators, is a severe blow to the confidence and career prospects of our amazing research community, which draws exceptional talent from across the United States and around the world. The American biomedical research establishment, fueled primarily by NIH grants, is a shining model for research, generating phenomenal advances to improve the health of Americans. It is essential that this model is not destroyed to achieve current political goals.

These are unprecedented times. Never before in our careers in biomedical research and public health have we seen the threats now imposed by recent US government decisions. The avalanche of worrisome news can be paralyzing, as it is difficult to know how to confront challenges coming from so many directions. It is critical that the ASTMH community does not lose sight of our primary goal, to reduce the worldwide burden of tropical infectious diseases and improve global health. Reaching out to elected representatives and engagement with non-scientific communities to broaden trust in science and explain the value of international health programs are vital. Now more than ever, the world needs us to work together to advocate for the importance of global health research and to improve health for all inside the United States and around the world.
